# Evaluation of the association of some circulating miRNA molecules in the metabolic syndrome

**DOI:** 10.5339/qmj.2024.71

**Published:** 2024-12-16

**Authors:** Weaam Gouda, Abd El-Haleem Ahmed, Abou El-Hamd H. Mohamed, Mohamed Abou-Ellail, Mie Afify, W.I. Hamimy, Mohamed D.E. Abdelmaksoud

**Affiliations:** ^1^Department of Biochemistry, Biotechnology Research Institute, National Research Centre, Dokki, Giza, Egypt; ^2^Department of Agricultural Biochemistry, Faculty of Agriculture and Natural Resources, Aswan University, Aswan, Egypt; ^3^Department of Chemistry, Faculty of Science, Aswan University, Aswan, Egypt; ^4^Department of Genetics, Faculty of Agriculture and Natural Resources, Aswan University, Aswan, Egypt; ^5^Anesthesia Department, Obesity Surgery Unit, Faculty of Medicine, Cairo University, Giza, Egypt *Correspondence: Weaam Gouda. Email: weaamgoudaali@gmail.com

**Keywords:** MiRNAs, metabolic syndrome, metabolically healthy obese, pre-metabolic syndrome

## Abstract

**Purpose:** The aim of this study was to investigate the possibility of including miRNA-371 and miRNA-143 in the early detection and diagnosis of the extent of the metabolic syndrome (MetS) in obese patients by measuring the expression of miRNA-143 and miRNA-371 in metabolically and pre-metabolically obese individuals and comparing the results with metabolically healthy obese controls. In addition, the study aimed to assess the correlation between the two types of miRNA and the criteria of MetS.

**Methods:** The expression levels of miRNA-143 and miRNA-371 were determined using quantitative real-time polymerase chain reaction (PCR) for 135 obese patients who were divided into the following three different categories based on metabolic criteria: 1) metabolic syndrome obese (MetS) group, 2) pre-metabolic syndrome obese (PreMetS) group, and 3) metabolically healthy obese (MHO) group.

**Results:** The results indicated a significant association of miRNA-143 and miRNA-371 with the MetS group compared with the PreMetS and MHO groups. As a result, the correlation analysis for these miRNAs revealed a large association with the results of the analysis for various factors, especially with regard to fasting glucose and lipid profiles in the MetS group.

**Conclusion:** There was an association between obesity and MetS. This study was able to establish the role of miRNA-371 and miRNA-143 molecules in metabolically obese individuals. Therefore, by tracking the regulatory pathway of these molecules and expanding the understanding of the process of regulation and interference with the various metabolic pathways, this study could provide a deeper analysis and understanding of the MetS in obesity and the molecular causes leading to it.

## INTRODUCTION

Metabolic syndrome (MetS) is the result of a group of metabolic disorders in the pathways of regulating blood sugar and building fats.^1^ Chronic low-grade inflammation and a prothrombotic condition leading to endothelial dysfunction and atherosclerosis are further characteristics of the MetS. This causes a number of diseases that differ in the nature of individuals and their lifestyles. However, they usually include cardiovascular diseases, insulin resistance, type 2 diabetes (T2D), high blood pressure (BP), and increased fat accumulation around the waist, leading to obesity.^2^ Based on available evidence from several populations, it can be stated that 20–30% of adults suffer from the MetS.^3^ As a result, over the last two decades, metabolic disorders have become increasingly widespread worldwide.^4^ In Africa, the overall frequency of MetS was 32.4%. Compared to children, adults had a significantly higher prevalence of MetS. Additionally, the prevalence of MetS was significantly higher in females than in males. Patients with tbl2D had the highest prevalence of MetS (66.7%), followed by those with coronary artery disease (55.2%) and cardiovascular disorders (48.3%). To overcome this trend, early prevention and treatment interventions are crucial.^5^ The criteria for the MetS have changed over time. The most current version was published in 2009 by the American Heart Association/National Heart, Lung, and Blood Institute and the International Diabetes Federation. This includes measurements of BP, triglycerides (TG), high-density lipoprotein (HDL) cholesterol, low-density lipoprotein (LDL) cholesterol, waist circumference (WC), and fasting glucose (FG). Any individual with three of the five anomalies would be eligible for the MetS.^6^ MetS is often diagnosed in people with a normal body mass index (BMI < 30 kg/m^2^) because the disorder is not only caused by elevated fat mass or BMI, where BMI is measured as weight in kilograms divided by height in meters squared. Likewise, some obese people (BMI ≥ 30 kg/m^2^) could not exhibit any MetS characteristics. Therefore, metabolically healthy obese (MHO) refers to this subgroup of obese patients. Since Vague established the term “MHO” in 1950 during his observational study, other definitions of the concept have evolved.^7^ As the transition from MHO to MetS is continuous, it is possible to identify the intermediate group of subjects who do not meet the requirements for the MetS but are not MHO. Scientists term this category of individuals the pre-metabolic syndrome (PreMetS) group.^8^

MiRNA molecules are defined as short molecules of non-coding RNA ranging from 18 to 22 nucleotides. They contribute to the regulation of transcription and transmission of chemical signals, as well as their contribution to the regulation of complex processes such as growth, development, and response to various stimuli.^9^ The difference in the levels of their organization results in some vital differences in humans and some diseases as a natural consequence of their contribution to the regulation of metabolic processes and energy production, as well as to the regulation of the construction of various compounds in cells.^10^ Studies have revealed a dizzying array of vital functions of these small molecules.^11^ The imbalance in the regulation of miRNA molecules directly contributes to the development of cardiovascular diseases by controlling the increase in blood cholesterol levels and the accumulation of TG in blood vessels.^12^ It also contributes to the presence of fatty liver disease, the growth of adipose tissue, and the accumulation of fat around the waist by regulating the growth of adipose tissue.^13^ MiRNA molecules interfere with the insulin signal transduction pathway, causing disruption of the chemical reaction process and a reduction in insulin receptors on the brightest cells, which contributes to the existence and development of insulin resistance, leading to tbl2D.^11^

MiRNA-143 and miRNA-371 are short non-coding RNA molecules. Many studies have shown that there are differences in the levels of gene expression of these two types of miRNA, which are usually associated with MetS and obesity, such as cardiovascular disease and tbl2D.^14^ Therefore, the present study aimed to evaluate the expression levels of miRNA-143 and miRNA-371 according to MetS/PreMetS/MHO categories and to assess their relationship with the prevalence of MetS.

## SUBJECTS AND METHODS

### Subjects

The study was conducted on 135 obese patients (BMI ≥ 30 kg/m^2^) from Obesity Unite, Kasr El-Aini Hospital. Before measurements, written informed consent was obtained from all members involved in this study. The Research Ethics Committee, National Research Centre, Egypt approved this research protocol and informed consent (registration no. 19-162). Patients who participated in the study were selected according to special criteria, such as the study participants were at least 18 years old and were obese, as determined by BMI according to the World Health Organization (WHO) classification. BMI is a convenient, although relatively arbitrary, method for measuring obesity.^15^ Recruitment of obese patients was restricted by the following criteria: abnormal liver function, abnormal thyroid function, renal diseases, cardiovascular disease, chronic lung disease, steroid treatment, or history of cancer.

After 8 hours of fasting, a blood sample was taken from each participant and divided into two parts. One part was collected on ethylenediamine tetraacetic acid (EDTA) to separate plasma for the investigation of miRNA-143 and miRNA-371 expression using quantitative real-time reverse transcription polymerase chain reaction (qRT-PCR). The other part was left to clot for 10 min and then centrifuged at 1000 × g for 5 min. The separated serum was used for determining the biochemical tests.

The obese patients were divided into three different categories based on BMI, WC, FG, lipid profile (total cholesterol (TC), TG, HDL, and LDL), and BP as follows. 1) Patients who met three or more of the following criteria were included in the metabolic syndrome (MetS) group: systolic blood pressure ≥ 130 mmHg or diastolic blood pressure ≥ 85 mmHg, or previously diagnosed high BP; WC ≥ 94 cm in males or ≥ 80 cm in females; TG level ≥ 150 mg/dL or treatment of high TG; HDL level < 50 mg/dL in females and < 40 mg/dL in males, or treatment of low HDL; and FG ≥ 100 mg/dL or treatment of high glucose concentration. 2) The PreMetS group did not meet all of the MetS criteria but met at least one (WC excluded). 3) Subjects in the MHO group did not meet any of the MetS criteria and did not receive treatment for hypolipemic, glucose-lowering, or antihypertensive conditions.

### Methods

#### Biochemical analyses

All the study participants underwent the following biochemical measurements: fasting blood glucose, lipid profile (TG, TC, LDL, and HDL), kidney function test (urea and creatinine), and liver function test (alanine transaminase (ALT) and aspartate transaminase (AST)). These assessments were conducted using standard clinical laboratory procedures and commercial kits used according to the manufacturer's instructions.

#### Isolation and purification of miRNA-143 and miRNA-371

This process was carried out by isolating RNA molecules from plasma samples taken from the participants in several steps as follows: 1) the separation and purification of total cell-free RNA, which primarily includes small RNAs, such as miRNAs, from small amounts (up to 200 μl) of plasma using the miRNeasy Plasma Kit (Qiagen Inc., Valencia, CA, USA. cat. no. 52304); 2) the concentration of micro-RNA was measured using NanoDrop and the extraction quality was evaluated at A260/A280; 3) reverse transcription (RT) for miRNAs to complementary deoxyribonucleic acid (cDNA) was performed using the miScript II RT Kit (catalog no. 218161) from Qiagen, Germany; 4) quantification of miRNAs was performed by qRT-PCR using target-specific miScript primers (Table 1) and the miScript SYBR Green PCR Kit (Qiagen, Germany, catalog no. 218073). The real-time PCR equipment PCR system 2700 (Applied Biosystems, USA) was used to perform qRT-PCR procedures. The PCR reaction conditions were 95°C for 1 min, then 40 cycles of 95°C for 15 s, 55°C for 30 s, and 72°C for 30 s. Each reaction was carried out in triplicate, with U6 as an endogenous miRNA control. The relative expressions of different miRNAs were assessed using the 2^− ΔΔCt^ method.^16^

#### Statistical analysis

SPSS 22.0 (IBM, USA) was used for statistical analysis throughout the present research. Additionally, PASS 11 was used for calculating the sample size and statistical power. Data are presented as mean ±  standard error (SE) using one-way ANOVA to examine variations among the three studied groups. Correlations between the expression levels of miRNAs and different parameters were identified using Pearson's correlation analysis, and linear regression analysis was performed to predict the risk factors for the MetS. Graphs using scatterplots were used to display the significant correlations, and boxplots were used to represent the relative expressions of miRNAs. *P* values were considered statistically significant at *p* ≤ 0.05.

## RESULTS

The study included a total of 135 obese patients with ages ranging from 19 to 65 years and mean age ±  SE = 38.9 ± 1.44. The results of the measurements differed significantly in each of the BMI, WC, FG, and lipid profiles (TC, TG, HDL, LDL) between different metabolic groups, as shown in Table 2, which indicated the presence of excessive obesity (BMI ≥ 40 kg/m^2^) in the two groups (MetS (47.47 kg/m^2^) and PreMetS (41.81 kg/m^2^)). In addition, there was a close increase in fasting blood sugar levels between the MetS and PreMetS groups. The lipid profile study revealed that both the PreMetS and MetS groups had significantly higher LDL, TG, and TC levels. The third group (MHO) had lower TC, TG, and LDL levels than its counterparts, but higher HDL levels. Depending on the analyses of urea and creatinine to measure kidney function and analyses of AST and ALT to measure liver function, the results were not significant between the three metabolic categories (MetS, PreMetS, and MHO), as shown in Table 2.

**Figure 1.**
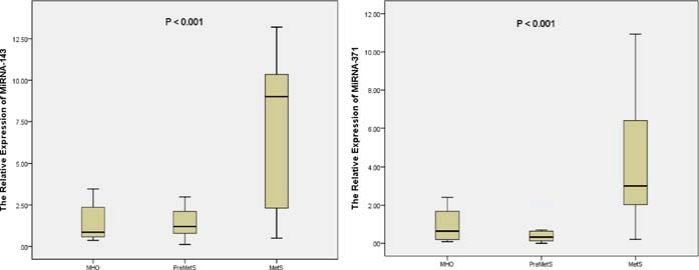
Relative expression of miRNAs (143 and 371) in obese patients according to the metabolic syndrome. MHO group: metabolically healthy obese patients (non-metabolic), PreMetS group: pre-metabolic syndrome obese patients, MetS group: metabolic syndrome obese patients.

When classifying the obese patients into different metabolic groups based on the previous measurements, the results of miRNA (143 and 371) expressions showed highly significant differences between the studied groups at a *p*-value of less than 0.001, as shown in Table 3 and Figure 1. The expression level of miRNA-143 was increased in the MetS group (6.74), while it was decreased in the PreMetS group (1.69) and the MHO group (1.67). Regarding the expression level of miRNA-371, the highest expression level was reported in the MetS group (3.79).

Table 4 shows that the results of the correlation analysis between BMI, metabolic indices, lipid profile (TC, TG, HDL, and LDL), and miRNAs (134 and 371) were not significant in the MHO group.

**Figure 2.**
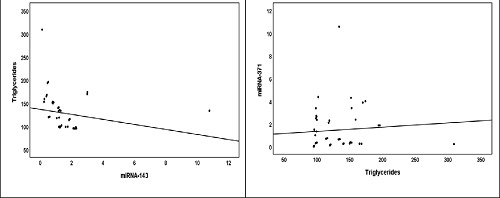
Correlations between miRNAs (143 and 371) and triglycerides in pre-metabolic syndrome.

In the PreMetS group, the correlations between miRNAs (143 and 371) and BMI, metabolic indices, and lipid profile are presented in Table 5 and Figure 2. The results showed a significant correlation between miRNA-143 and TG (*p* = 0.001, *r* = -0.641**), where a negative correlation was found, which expressed the inverse relationship between miRNA-143 and TG (an increase in miRNA-143 levels corresponds to a decrease in TG levels). However, there was no significant correlation between BMI, FG, TC, HDL, LDL, and miRNA-143. Regarding miRNA-371, there was a significant positive correlation between miRNA-371 and TG (*p* = 0.02, *r* = 0.473*), which expressed a direct relationship between miRNA-371 and TG so that increased TG levels were associated with increased levels of miRNA-371. However, there was no significant correlation between miRNA-371 and BMI, FG, TC, HDL, and LDL.

**Figure 3.**
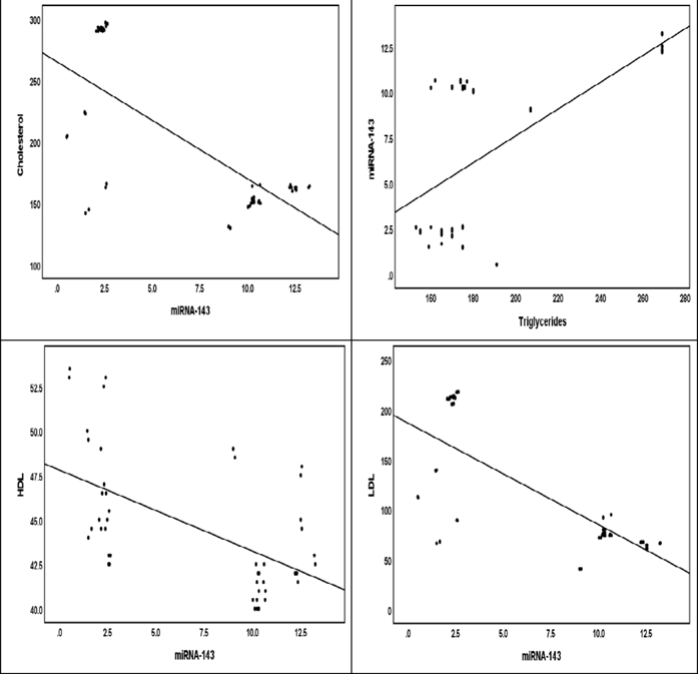
Correlations between miRNA-143 and lipid profiles in metabolic syndrome patients.

**Figure 4.**
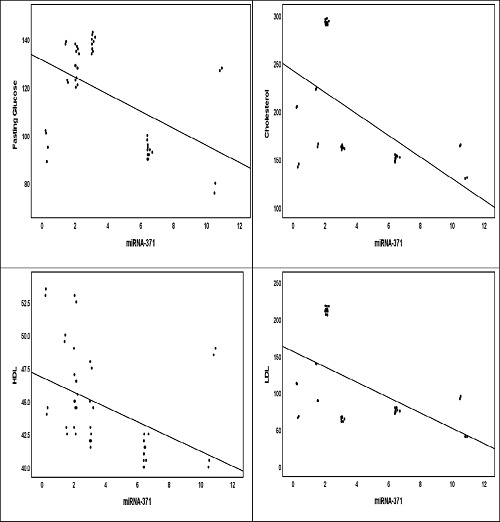
Correlations between miRNA-371 and lipid profiles in the MetS group.

The results of the correlation analysis between miRNA-143 and BMI were significant (*p* = 0.05), corresponding to a positive correlation (*r* = 0.397*), as shown in Table 6 and Figure 3. In addition, there was a significant positive association between miRNA-143 and TG (*p* = 0.00, *r* = 0.672**)**.** However, we found that there were negative correlations between miRNA-143 and HDL (*r* = -0.580**), LDL (*r* = -0.762**), and cholesterol (*r* = -0.734**), all of which were highly significant at a *p*-value of less than 0.01. The results of the correlation analysis of miRNA-371 showed a significant association with FG, TC, HDL, and LDL levels. These associations represented an inverse relationship (FG: *r* = -0.507**, TC: *r* = -0.544**, HDL: *r* = -0.458*, LDL: *r* = -0.499*), as increased expression of miRNA-371 corresponded to a decrease in FG, TC, HDL, and LDL levels (Table 6 and Figure 4).

Table 7 shows the results of the linear regression analyses of miRNA-143 and miRNA-371 with different parameters in the PreMetS and MetS groups. BMI, FG, and TG were found to be the risk factors for PreMetS in miRNA-143 and miRNA-371. However, BMI, TG, LDL, and HDL were the predictive risk parameters in the MetS group.

## DISCUSSION

MiRNAs are essential components of metabolism and play an important role in the major metabolism processes. Accordingly, they could potentially serve as therapeutic targets for the treatment of conditions such as obesity, cardiovascular diseases, and some metabolic disorders.^11^ MetS is a combination of several characteristics, including obesity, diabetes, dyslipidemia, hypertension, and cardiovascular diseases. As a result, it has been widely established that the MetS, which is closely associated with the increase in obesity and sedentary behavior, has become a global health concern.^19^ Consequently, in our study, obese patients were divided into three categories according to the presence of metabolic criteria such as MetS, PreMetS, and MHO. A good way to differentiate between the individuals who belonged to the MetS category was that they had different numbers of diseases associated with MetS such as high blood fats, tbl2D, and hypertension. The PreMetS group was categorized according to the presence of one of the symptoms of MetS, and the results of the MetS and PreMetS groups were compared with those of the MHO group. The results showed that the patients in the metabolic category had highly significant differences in blood sugar. This could be explained by the fact that people who are overweight, as those in the MetS group, usually have a clear imbalance in the amount of insulin in the blood, which usually leads to type 2 diabetes mellitus (T2DM), while people with normal metabolism can regulate blood sugar levels.^20^ Obese patients usually develop atherosclerotic diseases, which are due to the accumulation of fats and cholesterol in blood vessels, impeding blood flow and placing greater stress on the heart.^21^ This is evident from our results of the lipid profile analysis of the MetS group, which showed a large increase in TG and cholesterol. Therefore, this significant imbalance observed in the lipid analysis results is expected to lead to cardiovascular disease, which is a metabolic complication. In contrast, metabolically healthy obese individuals have less fat accumulation, and therefore obese individuals are still at higher risk of cardiovascular diseases and tbl2DM compared with metabolically healthy individuals of normal weight.^22^

Since miRNAs are degraded more slowly than mRNAs and proteins, they are more stable, sensitive, and valuable for tracking disease progression as well as for early diagnosis and risk assessment.^23,24^ Using integrative miRNA–gene pathway networks, it is possible to identify miRNA signatures associated with diseases. In fact, miRNAs have been shown to have an impact on a number of pathological processes, including metabolism.^25,26^ Elucidating the regulatory mechanism of miRNAs in cellular metabolism during pathological conditions as well as developing appropriate preventive strategies that target metabolic pathways are the fundamental steps in a potential intervention strategy that could alter metabolism in disorders.^11^ Therefore, the present study that examined the expressions of miRNA-371 and miRNA-143 could help in the management of MetS. By categorizing the patients according to the MetS, we could have a clear idea of the extent to which miRNA-371 and miRNA-143 are related to the different symptoms over time from PreMetS to the onset of various symptoms of MetS, which is evident from the results of the miRNA expression and correlation analyses. MiRNAs regulate energy balance and metabolic homeostasis by controlling various metabolic pathways.^27^ Obesity is a medical condition that results from the MetS, as it affects many vital functions and causes many adverse effects on the physical condition of an individual, reflecting the number of internal changes that occur in the essential processes.^28^ In this regard, our results indicated that there was a highly significant association between the expressions of miRNAs (371 and 143) and the MetS. This is due to the involvement of these miRNA molecules in the regulation of metabolic processes, especially the regulation of blood sugar and the growth of adipose tissue.^27^ Given the differences in miRNA-143 levels between the three groups, it was found that there was a higher expression in the MetS group compared with the PreMetS and MHO groups, leading to the possibility of developing tbl2D due to increased insulin resistance resulting from the interference of miRNA-143 in the insulin signal transduction pathway that causes a decrease in insulin receptors in cell membranes.^29^ Furthermore, previous studies reported that miRNA-143 is important for adipogenesis.^22,30^

The results of miRNA-371 showed that there was a significant increase in the MetS group compared with the PreMetS and MHO groups, but the increased level was less than its counterpart (miRNA-143). When studying the nature of the biological interference of miRNA-371 in metabolic processes, our results were consistent with previous research that found that the cause of a number of diseases is the presence of weight gain or disturbances in metabolism and diseases associated with obesity.^31,32^

Given the reasons for choosing the PreMetS obese group as part of our comparison, the aim is to know the extent of the association between the miRNA molecules 371 and 143 and the presence of one of the pathological symptoms associated with the metabolic condition in individuals with weight gain. This was highlighted by the significant results obtained from miRNAs and lipid profile correlations of the PreMetS group. In PreMetS obese, the results of the correlation analysis between miRNA-143 and TG were highly significant, which is due to hyperlipidemia, one of the major risk factors in obese, insulin resistance, and tbl2D patients.^33,34^ Although there was a positive correlation between the results of miRNA-371 and TG, this positive relationship was due to the close association between miRNA-371 and the regulation of adipose tissue function in the body.^35^ These findings are consistent with a previous study that found that lipid profiles are usually the first factor affected by weight gain and the development of insulin resistance.^36^ In comparison to the results of miRNA-143 and miRNA-371 and their association with various symptoms of obesity, the results showed that there were differences in the association with various pathological symptoms in the MetS group, such as the positive association between miRNA-143 and BMI, which is consistent with a previous study.^27^ In addition, the results of the correlation analysis with the FG level were significant. As suggested by previous research, the reason for this direct relationship lies in the regulatory behavior of miRNA-143 and its interference in disrupting the intracellular response to insulin signaling.^29^ Regarding lipid profiles, there was a significant correlation between the lipid profiles (cholesterol, HDL, and LDL) and miRNA-143 and miRNA-371, while there was an association between miRNA-143 and TG. This could be explained by the fact that miRNAs (143 and 371) strongly interfere with the processes of regulating adipose tissue in the body.^31^

## LIMITATIONS

We believe that these miRNAs may be more relevant than those detected in animal models and therefore may be more appropriate to provide insight into the prospective significance of the currently evaluated miRNAs in metabolic disorders, as all samples in the study were collected from human patients. One of the limitations is the small number of participants in the study. Therefore, larger research investigations involving a variety of subjects are necessary to clarify the roles of miRNA-143 and miR-371 in the development of MetS and associated disorders.

## CONCLUSION

MiRNAs associated with the MetS could play a critical role in managing the MetS. This opens opportunities for the use of miRNA-based approaches, has negligible harmful effects, and is well tolerated by patients. The present findings corroborated previous studies showing that miRNAs have a compensatory effect in the MetS. Therefore, this investigation revealed that the expressions of miRNA-143 and miRNA-371 were significantly associated with the MetS group compared with the PreMetS and MHO groups, suggesting the involvement of these miRNAs molecules in the regulation of metabolic processes. This finding opens new perspectives and highlights the role of the two types of miRNA in the etiology of MetS.

### Ethics approval

This study was approved by the Medical Ethics Committee of the National Research Centre (no. 19-162) and written informed consent was obtained from all participants.

### List of abbreviations

[Table tbl8]

### Competing interests

The authors declare that they have no conflict of interest.

### Funding

This work was supported by project grants from the National Research Centre (project no. 12060170), Egypt.

### Authors’ contributions

All the authors have accepted responsibility for the entire content of this submitted manuscript and approved the submission.

### Acknowledgments

We would like to thank the National Research Centre for the technical and financial support of the current research. We thank the patients and subjects who participated in this study.

## Figures and Tables

**Table 1:** Primers for miRNA-143, miRNA-371, and U6.

miR-143 (RT)	GTCGTATCCAGTGCAGGGTCCGAGGTATTCGCACTGGATACGACGAGCTACA	Ref. [17]

miR-143 (F)	ATGGTTCGTGGGTGAGATGAAGCACTG	

miR-143 (R)	GTGTCGTGGAGTCGGCAATTC	

U6 (RT)	CGCTTCACGAATTTGCGTGTCAT	

U6 (F)	GCTTCGGCAGCACATATACTAAAAT	

U6 (R)	CGCTTCACGAATTTGCGTGTCAT	

miR-371-5p RT	GTCGTATCCAGTGCAGGGTCCGAGGTGCACTGGATACGACAGTGCC	Ref. [18]

miR-371-5p F	TGCGGACTCAAACTGTGGGGGC	

miR-371-5p R	CCAGTGCAGGGTCCGAGGT	


**Table 2:** Laboratory and anthropometric measurements according to the metabolic category.

Parameter	MetS *n* = 47 Mean ± SE	PreMetS *n* = 40 Mean ± SE	MHO *n* = 48 Mean ± SE	*p*-value

Age (years)	42.4 ± 2.4	38.92 ± 2.4	33.71 ± 2.3	0.059

BMI (kg/m^2^)	47.47 ± 1.4	41.81 ± 1.6	37.797 ± 1.3	< 0.001

WC (cm)	129 ± 13.8	112.1 ± 14.2	104.7 ± 15.6	0.013

FG (mg/dL)	117.96 ± 4.2	105 ± 5.7	84.7 ± 1.9	< 0.001

TC (mg/dL)	203.64 ± 12.9	179.50 ± 6.6	158.65 ± 8.9	0.015

TG (mg/dL)	190.6 ± 8.3	131.4 ± 9.6	105.3 ± 5.5	< 0.001

HDL (mg/dL)	44.8 ± 0.77	49.46 ± 0.76	59.53 ± 0.84	< 0.001

LDL (mg/dL)	120.72 ± 13.3	103.75 ± 6.7	78.06 ± 9.3	0.029

Urea (mg/dL)	25.08 ± 0.37	24.32 ± 0.67	23.46 ± 0.53	0.140

Creatinine (mg/dL)	0.914 ± 0.022	0.939 ± 0.024	0.878 ± 0.032	0.264

AST (IU/L)	23.92 ± 0.53	24.92 ± 0.69	23.18 ± 0.68	0.183

ALT (IU/L)	23.8 ± 0.77	22.63 ± 0.48	24.35 ± 0.91	0.238


The ANOVA test was used for comparison between the three groups as mean ±  SE (standard error). The *p-values* in bold show a significant difference (*p* < 0.05).

BMI: body mass index, WC: waist circumference, FG: fasting glucose, TC: total cholesterol, TG: triglycerides, HDL: high-density lipoprotein cholesterol, LDL: low-density lipoprotein cholesterol, AST: aspartate transaminase, ALT: alanine transaminase.

MetS group: metabolic syndrome obese patients.

PreMetS group: pre-metabolic syndrome obese patients.

MHO group: metabolically healthy obese patients (non-metabolic).

**Table 3:** Expression levels of circulating miRNAs (143 and 371) in the different metabolic groups.

Parameter	Mean	SE	95% Confidence interval for mean	*p*-value

miRNA-143				

MetS	6.74	0.94	4.79–8.69	

PreMetS	1.69	0.42	0.82–2.56	< 0.001

MHO	1.67	0.53	0.54–2.81	

miRNA-371				

MetS	3.79	0.58	2.6–4.98	

PreMetS	1.1	0.46	0.15–2.04	< 0.001

MHO	0.96	0.22	0.48–1.43	


The ANOVA test was used for comparison between the three groups as mean and SE (standard error).

The *p* values in bold show a significant difference (*p* < 0.05).

MetS group: metabolic syndrome obese patients.

PreMetS group: pre-metabolic syndrome obese patients.

MHO group: metabolically healthy obese patients (non-metabolic).

**Table 4:** Correlations between miRNAs and body mass index, metabolic indices and lipid profiles in metabolically healthy obese patients.

	BMI	Fasting glucose	Cholesterol	Triglycerides	HDL	LDL

miRNA-143						

Pearson’s correlation	0.344	0.319	0.001	-0.197	0.127	0.012

Significance	0.177	0.212	0.998	0.448	0.628	0.962

miRNA-371						

Pearson’s correlation	0.158	-0.069	0.095	0.353	-0.353	0.082

Significance	0.544	0.792	0.717	0.165	0.164	0.755


**Table 5:** Correlations between miRNAs and body mass index, metabolic indices and lipid profiles in pre-metabolic syndrome obese patients.

	BMI	Fasting glucose	Cholesterol	Triglycerides	HDL	LDL

miRNA-143						

Pearson’s correlation	-0.054	0.154	-0.085	-0.641**	0.344	-0.036

Significance	0.802	0.473	0.694	0.001	0.100	0.869

miRNA-371						

Pearson’s correlation	0.341	-0.150	0.254	0.473*	-0.178	0.243

Significance	0.103	0.484	0.231	0.020	0.406	0.253


Bold values indicate statistically significant differences: *p < 0.05, **p < 0.01.

**Table 6:** Correlations between miRNAs and body mass index, metabolic indices and lipid profiles in metabolic syndrome obese patients.

	BMI	Fasting glucose	Cholesterol	Triglycerides	HDL	LDL

miRNA-143						

Pearson’s correlation	0.397*	-0.154	-0.734**	0.672**	-0.580**	-0.762**

Significance	0.050	0.462	0.000	0.000	0.002	0.000

miRNA-371						

Pearson’s correlation	0.345	-0.507**	-0.544**	-0.018	-0.458*	-0.499*

Significance	0.091	0.010	0.005	0.933	0.021	0.011


Bold values indicate statistically significant differences: *p ≤ 0.05, **p ≤ 0.001.

**Table 7:** Linear regression analysis of miRNA-143 and miRNA-371 with demographic and lipid profiles in pre-metabolic and metabolic syndrome groups.

	Pre-metabolic syndrome (*n* = 40)		Metabolic syndrome (*n* = 47)	

	β	*p*-value	β	*p*-value

miRNA-143				

Age	0.258	0.198	0.108	0.089

Sex	0.157	0.378	-0.151	0.214

BMI	-0.707	0.002	0.445	0.009

FG	0.607	0.027	-0.008	0.958

TG	-0.702	0.006	0.837	< 0.001

HDL	-0.344	0.175	-0.437	< 0.001

LDL	-0.157	0.428	-0.867	0.002

miRNA-371				

Age	0.086	0.626	-0.133	0.256

Sex	0.129	0.416	-0.237	0.295

BMI	0.986	< 0.001	1.115	< 0.001

FG	-0.934	< 0.001	0.419	0.142

TG	0.514	0.023	-1.295	< 0.001

HDL	-0.325	0.151	-0.490	0.006

LDL	0.037	0.835	-2.056	< 0.001


β: standardized coefficient beta. The*p* values in bold are considered significant (*p* < 0.05).

**Table tbl8:** 

ALT	Alanine Transaminase

AST	Aspartate Transaminase

BMI	Body Mass Index

FG	Fasting Glucose

HDL	High-Density Lipoprotein

LDL	Low-Density Lipoprotein

MetS	Metabolic Syndrome

MHO	Metabolically Healthy Obese

PreMetS	Pre-Metabolic Syndrome

qRT-PCR	Quantitative Real-Time Reverse Transcription PCR

T2D	Type 2 Diabetes

TC	Total Cholesterol

TG	Triglycerides

WC	Waist Circumference

WHO	World Health Organization


## References

[1] Lemos GDO, Torrinhas RS, Waitzberg DL (2023). Nutrients, physical activity, and mitochondrial dysfunction in the setting of metabolic syndrome. Nutrients.

[2] Bazmandegan G, Abbasifard M, Nadimi AE, Alinejad H, Kamiab Z (2023). Cardiovascular risk factors in diabetic patients with and without metabolic syndrome: A study based on the Rafsanjan cohort study. Sci Rep.

[3] Grundy SM (2008). Metabolic syndrome pandemic. Arterioscler Thromb Vasc Biol.

[4] Chew NWS, Ng CH, Tan DJH, Kong G, Lin C, Chin YH (2023). The global burden of metabolic disease: Data from 2000 to 2019. Cell Metab.

[5] Bowo-Ngandji A, Kenmoe S, Ebogo-Belobo JT, Kenfack-Momo R, Takuissu GR, Kengne-Ndé C (2023). Prevalence of the metabolic syndrome in African populations: A systematic review and meta-analysis. PLoS One.

[6] Alberti KGMM, Eckel RH, Grundy SM, Zimmet PZ, Cleeman JI, Donato KA (2009). Harmonizing the metabolic syndrome: A joint interim statement of the International Diabetes Federation Task Force on Epidemiology and Prevention; National Heart, Lung, and Blood Institute; American Heart Association; World Heart Federation; International Atherosclerosis Society; and International Association for the Study of Obesity. Circulation.

[7] Vague J (1956). The degree of masculine differentiation of obesities: A factor determining predisposition to diabetes, atherosclerosis, gout, and uric calculous disease. Am J Clin Nutr.

[8] Milewska EM, Szczepanek-Parulska E, Marciniak M, Krygier A, Dobrowolska A, Ruchala M (2022). Selected organ and endocrine complications according to BMI and the metabolic category of obesity: A single endocrine center study. Nutrients.

[9] Afsharmanesh MR, Mohammadi Z, Mansourian AR, Jafari SM (2023). A review of micro RNAs changes in T2DM in animals and humans. J Diabetes.

[10] Liu C, Feng H, Zhang L, Guo Y, Ma J, Yang L (2022). MicroRNA1433p levels are reduced in the peripheral blood of patients with gestational diabetes mellitus and influences pancreatic βcell function and viability. Exp Ther Med.

[11] Zong Y, Wang X, Cui B, Xiong X, Wu A, Lin C (2023). Decoding the regulatory roles of non-coding RNAs in cellular metabolism and disease. Mol Ther.

[12] Caliskan A, Crouch SA, Dangwal S

[13] Marketou M, Kontaraki J, Kalogerakos P, Plevritaki A, Chlouverakis G, Kassotakis S (2023). Differences in microRNA expression in pericoronary adipose tissue in coronary artery disease compared to severe valve dysfunction. Angiology.

[14] Liu J, Wang H, Zeng D, Xiong J, Luo J, Chen X (2023). The novel importance of miR-143 in obesity regulation. Int J Obes.

[15] Carter PJ, Taylor BJ, Williams SM, Taylor RW (2011). Longitudinal analysis of sleep in relation to BMI and body fat in children: The FLAME study. BMJ.

[16] Livak KJ, Schmittgen TD (2001). Analysis of relative gene expression data using real-time quantitative PCR and the 2^− ΔΔCt^ method. Methods.

[17] Zheng B, Xue X, Zhao Y, Chen J, Xu CY, Duan P (2014). The differential expression of microRNA-143,145 in endometriosis. Iran J Reprod Med.

[18] Liu RY, Diao CF, Zhang Y, Wu N, Wan HY, Nong XY (2013). miR-371-5p down-regulates pre mRNA processing factor 4 homolog B (PRPF4B) and facilitates the G1/S transition in human hepatocellular carcinoma cells. Cancer Lett.

[19] Huang Y, Yan Y, Xv W, Qian G, Li G, Zou H (2018). A new insight into the roles of MiRNAs in metabolic syndrome. Biomed Res Int.

[20] Tzenios N (2023). Obesity as a risk factor for different types of cancer. EPRA Int J Res Dev (IJRD).

[21] Perone F, Pingitore A, Conte E, Halasz G, Ambrosetti M, Peruzzi M, Cavarretta E (2023). Obesity and cardiovascular risk: Systematic intervention is the key for prevention. Healthcare (Basel).

[22] ENGIN AB, ENGIN A (2022). Adipogenesis-related microRNAs in obesity. ExRNA.

[23] Goguet-Rubio P, Klug RL, Sharma DL, Srikanthan K, Puri N, Lakhani VH (2017). Existence of a strong correlation of biomarkers and miRNA in females with metabolic syndrome and obesity in a population of West Virginia. Int J Med Sci.

[24] Chen X, Ba Y, Ma L, Cai X, Yin Y, Wang K (2008). Characterization of microRNAs in serum: A novel class of biomarkers for diagnosis of cancer and other diseases. Cell Res.

[25] Zeng X, Zhang X, Zou Q (2016). Integrative approaches for predicting microRNA function and prioritizing disease-related microRNA using biological interaction networks. Brief Bioinform.

[26] Wang L, Shang C, Pan H, Yang H, Zhu H, Gong F (2021). MicroRNA expression profiles in the subcutaneous adipose tissues of morbidly obese Chinese women. Obes Facts.

[27] Goncalves BS, Meadows A, Pereira DG, Puri R, Pillai S (2023). Insight into the inter-organ crosstalk and prognostic role of liver-derived microRNAs in metabolic disease progression. Biomedicines.

[28] Purnell JQ Definitions, classification, and epidemiology of obesity. In: Feingold KR, Anawalt B, Blackman MR, et al. (eds.). Endotext [Internet]. http://mdtext.com.

[29] Chen W, Chen Y, Hui T (2023). MicroRNA-143 interferes the EGFR-stimulated glucose metabolism to re-sensitize 5-FU resistant colon cancer cells via targeting hexokinase 2. J Chemother.

[30] Xie H, Lim B, Lodish HF (2009). MicroRNAs induced during adipogenesis that accelerate fat cell development are downregulated in obesity. Diabetes.

[31] Otsuka K, Nishiyama H, Kuriki D, Kawada N, Ochiya T (2023). Connecting the dots in the associations between diet, obesity, cancer, and microRNAs. Semin Cancer Biol.

[32] Thibeault K, Légaré C, Desgagné V, White F, Clément AA, Scott MS (2022). Maternal body mass index is associated with profile variation in circulating microRNAs at first trimester of pregnancy. Biomedicines.

[33] van Wijk JP, Halkes CJ, Erkelens DW, Castro Cabeza M (2003). Fasting and daylong triglycerides in obesity with and without type 2 diabetes. Metabolism.

[34] Björnson E, Adiels M, Taskinen MR, Borén J (2017). Kinetics of plasma triglycerides in abdominal obesity. Curr Opin Lipidol.

[35] Bork S, Horn P, Castoldi M, Hellwig I, Ho AD, Wagner W (2011). Adipogenic differentiation of human mesenchymal stromal cells is down-regulated by microRNA-369-5p and up-regulated by microRNA-371. J Cell Physiol.

[36] Khamseh ME, Malek M, Abbasi R, Taheri H, Lahouti M, Alaei-Shahmiri F (2021). Triglyceride glucose index and related parameters (triglyceride glucose-body mass index and triglyceride glucose-waist circumference) identify nonalcoholic fatty liver and liver fibrosis in individuals with overweight/obesity.

